# Prevalence of oral mucosal normal variations and lesions in a middle-aged population: a Northern Finland Birth Cohort 1966 study

**DOI:** 10.1186/s12903-020-01351-9

**Published:** 2020-12-09

**Authors:** Ulla-Maija Oivio, Paula Pesonen, Merja Ylipalosaari, Arja Kullaa, Tuula Salo

**Affiliations:** 1 Cancer and Translational Medicine Research Unit, Faculty of Medicine, University of Oulu, P.O. Box 5281, 90014 Oulu, Finland; 2 Infrastructure for Population Studies, Faculty of Medicine, University of Oulu, P.O. Box 5000, 90014 Oulu, Finland; 3 Research Unit of Oral Health Sciences, University of Oulu, P.O. Box 5281, 90014 Oulu, Finland; 4 Institute of Dentistry, Faculty of Health Sciences, University of Eastern Finland, P.O. Box 1627, 70211 Kuopio, Finland; 5 Department of Oral Diagnostics, Educational Dental Clinic, Kuopio University Hospital, Yliopistonranta 1 C, 70210 Kuopio, Finland; 6 Medical Research Center Oulu, Oulu University Hospital and University of Oulu, P.O. Box 5281, 90014 Oulu, Finland; 7 Institute of Oral and Maxillofacial Disease, University of Helsinki, 00014 Helsinki, Finland; 8 HUSLAB, Department of Pathology, Helsinki University Hospital, Helsinki, Finland

**Keywords:** NFBC, Cross-sectional study, Middle-aged population, Oral mucosa, Normal variations and lesions, Oral lichen planus, Oral lichenoid reaction, Prevalence, Leukoplakia

## Abstract

**Background:**

In this cross-sectional study we investigated the oral mucosal changes in a middle-aged Finnish population. We analyzed the prevalence of potentially malignant disorders and the influence of smoking, snuff and alcohol use on the mucosal changes.

**Methods:**

Of the 12,068 members of the NFBC 1966, a total of 1961 participants (16.2%) constituted the study population. Mucosal changes were diagnosed and photographed by seven general dentists, and two specialists re-analyzed all the diagnoses based on the documentation Cross-tabulation with Chi-square tests and logistic regression analysis were used to analyze the data.

**Results:**

Of the participants, 10.5% had some mucosal changes, of which 81.8% were diagnosed as oral mucosal lesions (OML) and 18.2% as normal variations. Of the normal variations, the most common were Fordyce granules (1.2%), fissured tongue (1.1%) and geographic tongue (0.9%). The most common OMLs were white lesions (6.5%), of which oral lichen planus (OLP) and lichenoid reactions (OLR), grouped as oral lichenoid diseases, were present in 3.5%, males more often (3.8% vs*.* 3.1%). OLP was found in 1.5% of all participants, females more often (1.8% vs*.* 1.2%), while OLR was more common in males (2.7% vs. 1.3%). Leukoplakia was identified in 0.5% of the population; twice more often in males (0.6% vs. 0.3%). Erythroplakia was not found. Current smokers had higher risk for oral mucosal changes than former or non-smokers (OR 3.0, 95% CI 2.11–4.28), and snuff, used occasionally or regularly, also raised the risk (OR 2.6, 95% CI 1.48–4.70).

**Conclusions:**

In the middle-aged northern Finland population, 4% of OMLs were potentially malignant disorders, including OLR (2%), OLP (1.5%) and leukoplakia (0.5%). In particular, smoking and snuff use increased the risk for having any oral mucosa changes.

## Introduction

The prevalence of oral mucosal changes ranges between 10.8 and 61.6% in various populations [1–6]. Differences in the reported prevalence can be explained by study protocol, participant individual selection, genetics, age, and sex, as well as local and general risk factors in the study population [1, 2]. Oral mucosal changes can be divided by clinical features into the following major groups: normal variations and oral mucosal lesions (OML), including vesiculobullous lesions, ulcerative conditions, white lesions, red-blue lesions, pigmented lesions, verruca-papillary lesions, tongue and buccal mucosa swellings, gingival swellings, palatal swellings and floor of mouth swellings [7].

White lesions are relatively common in the oral cavity, of which some are related to mucocutaneous diseases [8]. Manifestation of oral lichen planus (OLP) is variable, being typically symmetrical white and reticular striae [7–10]. Based on a recent systematic review, the malignant transformation of OLP occurs in 1.4% of cases [11]. Around 60–80% of OLP patients are reported to be women [9, 10], and the typical age of its onset is 30–60 years [7, 9, 10]. Oral lichenoid reaction (OLR) differs clinically and/or histopathologically from OLP, usually occurring unilaterally [12, 13]. OLR may be caused by several factors, such as drugs, trauma and dental restorative materials [13], and 3.8% of OLRs are shown to transform into cancer [11]. OLP and OLR can clinically be grouped together and termed as oral lichenoid diseases (OLD) [12, 14].

Tobacco, snuff and chronic irritation often cause white hyperkeratosis [7], but leukoplakia is defined as a white patch or plaque that cannot be scratched off or clinically considered to be caused by any traumatic irritation from any other disease [7, 15, 16]. Tobacco use may induce leukoplakia [16, 17]. Malignant transformation of leukoplakia has recently been reported to be 9.5% [11]. Oral leukoplakia, OLP, OLR, oral submucous fibrosis and erythroplakia all belong to a group of oral potentially malignant disorders [11, 16].

The incidence of OMLs increases with age, partially due to physiological changes in the oral cavity but also due to the sustained impact of risk habits. Decreased saliva flow and long-lasting effects of local and systemic factors, such as alcohol intake, smoking, snuff and drug use predispose individuals to various lesions which are not present in children, unlike some normal mucosal variations, such as geographic tongue, which already exist among youth [3, 7, 18].

The main purpose of this study was to screen all mucosal changes in the Northern Finland middle-aged (45–47-year-old) participants and to evaluate their association with smoking, use of snuff and alcohol intake. To the best of our knowledge, this is the only study carried out on the prevalence of oral mucosal changes in a Finnish population that was selected only for the year of birth (1966), but not for any other factors.

## Materials and methods

### Study population

The study population is part of the longitudinal Northern Finland Birth Cohort 1966 (NFBC 1966) [20]. The entire cohort included 12,055 mothers whose expected delivery was in the year 1966. Altogether 12,231 children were born, 12,058 of whom were born alive. NFBC 1966 data have been collected with health questionnaires and clinical examinations at the ages of around 1, 14, 31 and 46 years. Since the 46-years clinical examination was performed between April 2012 and June 2013, and depending on the month the examinee was born, their age ranged from 45 to 47 years. Health questionnaires and written data from the oral mucosal clinical examination and pictures of 1,961 participants were also collected; see details below [20].

### Health questionnaires

In connection with the 46-year examination, all cohort members were sent the postal questionnaires, which they filled in either on the internet or on paper. The questionnaire covered general health, sex, alcohol intake, smoking and use of snuff. Following the questionnaire, an invitation to a clinical examination was sent to all cohort participants.

Smoking status was assessed in the postal questionnaire as follows: “Have you ever smoked in your life?” (no, yes, I started at the age of XX), “Have you smoked regularly?” (no, yes, I have smoked regularly for a total of XX years), “If you have quit smoking, what age were you when you stopped?”, “Do you smoke currently?” (7 days a week, 5–6 days a week, 2–4 days a week, 1 day a week, occasionally, not at all). Usage of snuff was assessed as follows: “Do you use snuff currently?” (no, occasionally, regularly).

Alcohol consumption was queried in the questionnaire as follows: “How often do you usually drink beer, cider, long drinks or wine, fortified wines, homemade wines or hard liquors?” (never, less than once a year, a couple times a year, 3–4 times a year, once every 2 months, once a month, a couple times a month, once a week, a few times a week, daily), “How much beer, cider or long drinks do you usually drink on one occasion? (1 bottle = 1/3 L)” (less than 1 bottle, 1 bottle, 2 bottles, 3 bottles, 4–5 bottles, 6–9 bottles, 10–14 bottles, 15 bottles or more, I don’t drink any of these beverages), “How much wine, fortified wine or homemade wine do you usually drink on one occasion? (half glass, one glass (= 16 cl), a couple of glasses, about half of the bottle (large bottle = ¾ l), a bit more than a large bottle, about one large bottle, one or two large bottles, more than two large bottles, I don’t drink wine), “How much hard liquor do you usually drink on one occasion? (less than 1 tavern portion (less than 4 cl), 1 tavern portion (about 4 cl), 2 tavern portions, 3–4 tavern portions, 5–6 tavern portions, 7–10 tavern portions, about a half-litre bottle, more than a half-litre bottle, I don’t drink hard liquor).

### Oral examination

In the 46-year follow-up survey, altogether 3150 persons currently living in the city of Oulu or within a radius of 100 km were asked to participate in the oral examination, including oral mucosa and dental examination. Of the original subgroup, a total of 1961 persons (62.3%) attended the clinical oral examination and gave their consent to use the data; these persons comprised the present study population. The clinical oral examination was performed between April 2012 and June 2013.

The oral examination was conducted by seven general dentists during the period 2012–2013. All examiners used a standard clinical dental examination protocol. They were trained and calibrated by Oulu University dental clinic teachers, not including oral medicine specialists, before the examination and at the middle of the study period. All examinations were made in the Oulu University dental clinic with modern supplies and optimal light. Clinical examination of the oral cavity included a careful and systematic visual inspection of the whole oral cavity. Dentists also made a palpation for all areas of the oral mucosa. Biopsy was not taken. All oral mucosal changes were written documented and photographed during the clinical examinations. All examiners used the same type of camera (CANON 600D 60 mm Lens Macro 1:2,8, Flash Sigma EM-140 DG).

### Diagnoses for the mucosal changes

Afterwards, oral pathologist (TS) and oral mucosal disease specialists (AK, TS) simultaneously re-analysed all the pictures and, based on them and on the documentation by the general dentists, they provided consensus diagnoses for all the findings. Specialists disagreed in 24.3% of the lesions and agreed in 54.8% of the proposed diagnosis by the general dentists. Of the documented lesions, 20.9% had no proposed diagnosis by the general dentists and were diagnosed afterwards based documentation and pictures by two specialists. The total number of examinees who had oral mucosal lesions was 206. There were no false-positive cases.

### Statistical analysis

The study population was described using frequencies and percentages with the categorical variables gender, smoking, snuff use and alcohol intake as shown in Table 1. Smoking was categorised as current smokers, former smokers and non-smokers, and snuff usage as snuff-users and non-users. The consumption of alcohol (g/day) was calculated based on the questions concerning alcohol usage. Alcohol intake was categorised such that the heavy user limit for females was 20 g/day and for males 30 g/day.

**Table 1:** Characteristics of the study population

	Total	Male	Female
	n (%)	n (%)	n (%)
**Tobacco**			
Current smoker	343 (18.3)	167 (19.2)	176 (17.5)
Former smoker	534 (28.5)	291 (33.4)	243 (24.2)
Non-smoker	999 (53.3)	413 (47.4)	586 (58.3)
**Snuff**			
Regular user	31 (1.6)	30 (3.4)	1 (0.1)
Occasional user	39 (2.1)	38 (4.3)	1 (0.1)
Non-user	1820 (96.3)	810 (92.3)	1010 (99.8)
**Alcohol**			
Heavy user	232 (12.2)	150 (17.0)	82 (8.0)
Normal user	1673 (87.8)	732 (83.0)	941 (92.0)

The number of mucosal lesions per person was calculated, and lesions and diseases were categorised into 11 main groups based on the Regezi et al. textbook [7] as following: no changes, normal variations, vesiculobullous lesions, ulcerative conditions, white lesions, red-blue lesions, pigmented lesions, verruca-papillary lesions, connective tissue lesions (CTL), divided by region into tongue and buccal mucosal swellings, gingival swellings and floor of mouth swellings.

Cross-tabulation with Chi-square or Fisher’s exact test was used to compare the numbers of mucosal lesions, submucosal swellings, oral mucosal variations and mucosal diseases between genders. Additionally, the association between lesions and smoking within gender as well as association between lesions and gender within smoking have been analysed using cross-tabulation with Chi-square or Fisher’s exact test. Logistic regression analysis was used to present crude odds ratios (OR) and their 95% confidence intervals (CI) when comparing current and former smokers with non-smokers, snuff users with non-users and heavy alcohol users with moderate users or non-users. The adjusted logistic regression model was executed including smoking, snuff use and alcohol in the same model.

Differences between groups were considered statistically significant at *p*-values < 0.05. All analyses were performed using SPSS (version 24.0; SPSS, Inc., Chicago, IL, USA).

## Results

### Characteristics of the study population

The total number of clinically examined participants was 1961; women comprised 53.5% (n = 1050) of the population. The mean age was 46 years. The number of participants who answered the questions concerning smoking was 1876 (95.7%). We divided smokers into three categories: current smokers (18.3%), former smokers (28.5%) and non-smokers (53.3%). Concerning snuff use, 1890 participants (96.4%) answered the questions: 1012 females (96.3%) and 878 males (96.3%). The proportion of snuff users was 3.7%, of which 0.2% were females and 7.7% males of the entire population. Concerning alcohol intake, 1905 participants (97.1%) answered the questions: 882 males (46.3%) and 1023 females (53.7%). In this study, 8.0% of females and 17.0% of males were heavy users (Table 1).

### Prevalence of oral mucosal normal variations and lesions

Of the participants (n = 1961), 10.5% (n = 206) had some mucosal changes, slightly more in men (n = 108). One change was registered in 7.4% (n = 145), two in 2.8% (n = 54) and three in 0.4% (n = 7) of the participants. Males more often than females had two changes (3.7%, n = 34 vs. 1.9%, n = 20; *p* = 0.014).

Altogether, 55 different kinds of changes were recorded; 81.8% were diagnosed as OMLs and 18.2% belonged to the normal variations category. When we categorised lesions into the main groups, the most common group was white lesions (6.5%, n = 128), followed by normal variations (3.3%, n = 64) and red-blue lesions (1.3%, n = 25). White lesions were more often diagnosed in males (8.1% vs. 5.1%; *p* = 0.008), whereas pigmented lesions were more common in females (1.0% vs. 0.3%; *p* = 0.090). Normal variations were seen more often in males (4.1%, n = 37; *p* = 0.064) (Table 2).

**Table 2:** Oral mucosal normal variations and lesions

	Total	Male	Female	*p-*value
	n (%)	n (%)	n (%)	
No changes	1755 (89.5)	803 (88.1)	952 (90.7)	0.069
Normal variations	64 (3.3)	37 (4.1)	27 (2.6)	0.064
Vesiculobullous lesions	1 (0.1)	1 (0.1)	0 (0.0)	0.465
Ulcerative lesions	4 (0.2)	3 (0.3)	1 (0.1)	0.343
White lesions	128 (6.5)	74 (8.1)	54 (5.1)	0.008
Red-blue lesions	25 (1.3)	12 (1.3)	13 (1.2)	0.876
Pigmented lesions	13 (0.7)	3 (0.3)	10 (1.0)	0.090
Verruca-papillary lesions	3 (0.2)	2 (0.2)	1 (0.1)	0.600
CTL: tongue and buccal mucosal swellings	18 (0.9)	9 (1.0)	9 (0.9)	0.762
CTL: gingival swellings	5 (0.3)	3 (0.3)	2 (0.2)	0.668
CTL: floor of mouth swellings	1 (0.1)	0 (0.0)	1 (0.1)	1.000

CTL = connective tissue lesions

The most common normal variations were the following: Fordyce granules 1.2% (n = 24), fissured tongue 1.1% (n = 22), geographic tongue 0.9% (n = 18) and hairy tongue 0.2% (n = 3) (Table 3) [3, 6].

**Table 3:** Prevalence of normal oral mucosal variations and their distribution by sex

	Total	Male	Female	*p-*value
	n = 1961	n = 911	n = 1050	
	n (%)	n (%)	n (%)	
Fordyce granules	24 (1.2)	21 (2.3)	3 (0.3)	< 0.001
Fissured tongue	22 (1.1)	8 (0.9)	14 (1.3)	0.340
Geographic tongue	18 (0.9)	6 (0.7)	12 (1.1)	0.262
Hairy tongue	3 (0.2)	2 (0.2)	1 (0.1)	0.600
Linea alba	2 (0.1)	1 (0.1)	1 (0.1)	1.000
Erythema migrans (lip)	1 (0.1)	0 (0.0)	1 (0.1)	1.000
Exostosis	1 (0.1)	0 (0.0)	1 (0.1)	1.000
Melanotic macule	1 (0.1)	0 (0.0)	1 (0.1)	1.000
Mandible exostosis	1 (0.1)	1 (0.1)	0 (0.0)	0.465
Dry lips	1 (0.1)	1 (0.1)	0 (0.0)	0.465

Of the oral mucosal lesions, the most common findings were OLR 2.0% (n = 39), OLP 1.5% (n = 29), hyperkeratosis 0.7% (n = 14), fibroma 0.7% (n = 13), snuff-related lesions 0.5% (n = 10), amalgam pigmentation 0.4% (n = 7), median rhomboid glossitis 0.4% (n = 7), hemangioma 0.3% (n = 6), ulcer 0.3% (n = 6) and candidiasis 0.3% (n = 5). Leukoplakia was registered in only 0.5% (n = 9) of all participants (n = 1961) (Table 4).

**Table 4:** Prevalence of oral mucosal lesions and their distribution by sex

	Total	Male	Female	*p-*value
	n = 1961	n = 911	n = 1050	
	n (%)	n (%)	n (%)	
OLR	39 (2.0)	25 (2.7)	14 (1.3)	0.026
OLP	29 (1.5)	11 (1.2)	18 (1.8)	0.354
Hyperkeratosis	14 (0.7)	9 (1.0)	5 (0.5)	0.179
Fibroma	13 (0.7)	6 (0.7)	7 (0.7)	0.983
Snuff-related lesion	10 (0.5)	10 (1.0)	0 (0.0)	< .001
Amalgam pigmentation	7 (0.4)	1 (0.1)	6 (0.6)	0.131
Median rhomboid glossitis	7 (0.4)	4 (0.4)	3 (0.3)	0.711
Candidiasis + thrush	6 (0.3)	5 (0.5)	1 (0.1)	0.103
Hemangioma	6 (0.3)	3 (0.3)	3 (0.3)	1.000
Ulcer	6 (0.3)	3 (0.3)	3 (0.3)	1.000
Hematoma	5 (0.3)	2 (0.2)	3 (0.3)	1.000
Rhagards	4 (0.2)	2 (0.2)	2 (0.2)	1.000
Hyperpigmentation	3 (0.2)	1 (0.1)	2 (0.2)	1.000
Leukoplakia	9 (0.5)	6 (0.7)	3 (0.3)	0.318
Papilloma	3 (0.2)	2 (0.2)	1 (0.1)	0.600
Petechia	3 (0.2)	2 (0.2)	1 (0.1)	0.600
Foliate papilla hyperplasia	2 (0.1)	0 (0.0)	2 (0.2)	0.502
Tongue papilla atrophy	2 (0.1)	0 (0.0)	2 (0.2)	0.502
Oral mucocele/ranula	1 (0.1)	1 (0.1)	0 (0.0)	0.465
Linea alba	2 (0.1)	1 (0.1)	1 (0.1)	1.000
Erythema	2 (0.1)	1 (0.1)	1 (0.1)	1.000
Fistula	2 (0.1)	1 (0.1)	1 (0.1)	1.000
Tobacco hyperpigmentation	2 (0.1)	1 (0.1)	1 (0.1)	1.000
Aphthous ulcer	2 (0.1)	1 (0.1)	1 (0.1)	1.000
Gingiva hyperplasia	2 (0.1)	2 (0.2)	0 (0.0)	0.216
Prosthesis stomatitis	1 (0.1)	0 (0.0)	1 (0.1)	1.000
Mucosal burn	1 (0.1)	0 (0.0)	1 (0.1)	1.000
Glandula sublingualis swelling	1 (0.1)	0 (0.0)	1 (0.1)	1.000
Salivary calculus	1 (0.1)	0 (0.0)	1 (0.1)	1.000
Papular hyperplasia	1 (0.1)	0 (0.0)	1 (0.1)	1.000
Tongue papule	1 (0.1)	0 (0.0)	1 (0.1)	1.000
White drug reaction (antibiotics)	1 (0.1)	0 (0.0)	1 (0.1)	1.000
Cheek mucosa hyperplasia	1 (0.1)	0 (0.0)	1 (0.1)	1.000
Bite trauma	1 (0.1)	1 (0.1)	0 (0.0)	0.465
Cobblestone hyperplasia	1 (0.1)	1 (0.1)	0 (0.0)	0.465
Desquamative gingivitis	1 (0.1)	1 (0.1)	0 (0.0)	0.465
Hairy leukoplakia	1 (0.1)	1 (0.1)	0 (0.0)	0.465
Herpes simplex infection	1 (0.1)	1 (0.1)	0 (0.0)	0.465
Nicotine stomatitis	1 (0.1)	1 (0.1)	0 (0.0)	0.465
Non-homogenous leukoplakia	1 (0.1)	1 (0.1)	0 (0.0)	0.465
Palatal ulcer	1 (0.1)	1 (0.1)	0 (0.0)	0.465
Papilla incisor hyperplasia	1 (0.1)	1 (0.1)	0 (0.0)	0.465
Redness of papilla incisor	1 (0.1)	0 (0.0)	1 (0.1)	1.000
Solar cheilitis	1 (0.1)	1 (0.1)	0 (0.0)	0.465
Tongue hypertrophy	1 (0.1)	1 (0.1)	0 (0.0)	0.465

The two most common lesions, OLR and OLP, grouped together into OLD, were found in 3.5% of the study population: 3.1% in females and 3.9% in males. OLP was slightly more common among females (1.8% vs. 1.2%; *p* = 0.354), as was hyperpigmentation associated with amalgam (0.6% vs. 0.1%; *p* = 0.131). Males had OLR significantly more often (2.7% vs. 1.3%; *p* = 0.026), as well as hyperkeratosis (1.0% vs. 0.5%; *p* = 0.179). Additionally, Fordyce granules (2.3% vs. 0.3%; p < 0.001) and snuff-related lesions (1.0% vs. 0.0%; *p* = 0.001) were more common in males than females (Tables 3, 4).

### Lesions related to tobacco product use

In current smokers, OLP (3.5%, *p* = 0.001), OLR (4.1%, *p* = 0.016), hyperkeratosis (1.7%, *p* = 0.007) and snuff-related lesions (1.5%, *p* = 0.004) were more common than in non- or former smokers. Of all the current smokers with OLP, 75% (n = 9, *p* = 0.095) were females. OLR was more common in current smokers (4.1%: males 4.8% and females 3.4%), followed by former smokers (1.7%: males 2.4% and females 0.8%) and non-smokers (1.6%: males 2.4% and females 1.0%). Similarly, hyperkeratosis was more common in current smokers (1.7%, n = 6), compared to former (0.7%, n = 4) and non-smokers (0.2%, n = 2) (*p* = 0.007). Among males, hyperkeratosis was detected in 2.4% (n = 4) of current smokers, 0.3% (n = 1) of former and 0.5% (n = 2) of non-smokers (*p* = 0.046). Among females, hyperkeratosis was detected in 1.1% (n = 2) of current smokers, 1.2% (n = 3) of former and in no non-smokers (*p* = 0.029). Hyperpigmentation (0.6%, n = 2; *p* = 0.033) was only found in current smokers. Snuff-related lesions were present in 1.5% (n = 5) of current smokers, 0.7% (n = 4) of former smokers and 0.1% (n = 1) of non-smokers (*p* = 0.004). All snuff-related lesions were found in males (2.4%, n = 4, *p* = 0.013). Leukoplakia was found in 9 (0.5%) of all participants, and 5 (1.5%) of them were current smokers, 2 (0.4%) were former and 2 (0.2%) were non-smokers (*p* = 0.024) (Table 5).

**Table 5:** Lesions associated with smoking and sex

	Current smoker	Former smoker	Non-smoker	*p-*value^a^
	n = 343	n = 534	n = 999	
	n (%)	n (%)	n (%)	
**OLP**				
Male	3 (1.8)	3 (1.0)	3 (0.7)	0.500
Female	9 (5.1)	2 (0.8)	6 (1.0)	0.002
Total	12 (3.5)	5 (0.9)	9 (0.9)	0.001
*p-*value^b^	0.095	1.000	0.743	
**OLR**				
Male	8 (4.8)	7 (2.4)	10 (2.4)	0.255
Female	6 (3.4)	2 (0.8)	6 (1.0)	0.070
Total	14 (4.1)	9 (1.7)	16 (1.6)	0.016
*p-*value^b^	0.518	0.192	0.083	
**Leukoplakia**				
Male	3 (1.8)	2 (0.7)	1 (0.2)	0.092
Female	2 (1.1)	–	1 (0.2)	0.125
Total	5 (1.5)	2 (0.4)	2 (0.2)	0.024
*p-*value^b^	0.678	0.503	1.000	
**Hyperkeratosis**				
Male	4 (2.4)	1 (0.3)	2 (0.5)	0.046
Female	2 (1.1)	3 (1.2)	0 (0.0)	0.029
Total	6 (1.7)	4 (0.7)	2 (0.2)	0.007
*p-*value^b^	0.374	0.335	0.171	
**Hyperpigmentation**				
Male	1 (0.6)	0 (0.0)	0 (0.0)	0.192
Female	1 (0.6)	0 (0.0)	0 (0.0)	0.175
Total	2 (0.6)	0 (0.0)	0 (0.0)	0.033
*p-*value^b^	1.000	–	–	
**Snuff-related lesion**				
Male	5 (3.0)	4 (1.4)	1 (0.2)	0.013
Female	0 (0.0)	0 (0.0)	0 (0.0)	–
Total	5 (1.5)	4 (0.7)	1 (0.1)	0.004
*p-*value^b^	0.027	0.130	0.413	
**Geographic tongue**				
Male	0 (0.0)	1 (0.3)	5 (1.2)	0.341
Female	2 (1.1)	0 (0.0)	10 (1.7)	0.089
Total	2 (0.6)	1 (0.2)	15 (1.5)	0.031
*p-*value^b^	0.499	1.000	0.526	
**Fordyce granules**				
Male	2 (1.2)	4 (1.4)	13 (3.1)	0.178
Female	1 (0.6)	0 (0.0)	2 (0.3)	0.555
Total	3 (0.9)	4 (0.7)	15 (1.5)	0.364
*p-*value^b^	0.614	0.130	< 0.001	

^a^Comparation between smoking

^b^Comparation between sexes

Of normal variations, geographic tongue was seen in 1.5% (n = 15) of non-smokers (*p* = 0.031). In females, 1.1% (n = 2) of current smokers, no former smokers and 1.7% (n = 10) of non-smokers had geographic tongue (*p* = 0.089). In males, 1.2% (n = 5) of non-smokers had geographic tongue. Fordyce granules were more common in non-smokers (1.5%, n = 15), particularly in males (3.1%, n = 13, p < 0.001), compared to current smokers (0.9%, n = 3) or former smokers (0.7%, n = 4) (*p* = 0.364) (Table 5).

### Odds ratios and confidence intervals

When we compared non-smokers with current smokers and former smokers, the OR for current smokers was 3.0 (95% CI 2.11–4.28) for any mucosal changes. Use of snuff, occasionally or regularly, raised the risk of having mucosal changes (OR 2.6, 95% CI 1.48–4.70). Alcohol intake affected the risk for mucosal changes (OR 1.3, 95% CI 0.82–1.91). When we included smoking, snuff use and alcohol intake in the same adjusted model, the smoking and snuff remained significant (Table 6).

**Table 6:** Crude ORs and 95% CIs for any mucosal changes or diseases in association with smoking, snuff and alcohol comparing current smokers and former smokers to non-smokers, snuff users to non-snuff users and alcohol heavy users to normal users

	Crude OR (95% CI)	*p*-value	Adjusted OR (95% CI)	*p*-value
**Smoking**				
Current smoker	3.0 (2.11–4.28)	< 0.001	2.9 (2.03–4.18)	< *0.001*
Former smoker	1.3 (0.93–1.93)	0.120	1.3 (0.89–1.87)	0.178
Non-smoker (reference)	1.0		1.0	
**Snuff**				
Use occasionally or regularly	2.6 (1.48–4.70)	0.001	2.4 (1.31–4.36)	*0.005*
Non-snuff user (reference)	1.0		1.0	
**Alcohol**				
Heavy user	1.3 (0.82–1.91)	0.290	0.9 (0.59–1.43)	0.703
Normal user (reference)	1.0		1.0	

## Discussion

In this cross-sectional study, we investigated the prevalence of oral mucosal changes in the Northern Finland Birth cohort (1966) of 1961 participants at the age of 46 years. The overall prevalence of any mucosal changes was 10.5%, of which 4% were grouped as potentially malignant disorders: OLD was found in 3.5% and leukoplakia in 0.5% of the study population. Unlike alcohol intake, both current smoking and snuff use significantly increased the number of mucosal changes. In general, males had mucosal lesions more often than females, related to their drinking, smoking and snuff use habits.

The final diagnoses collected were based on documentation by a general dentist examiner that was later simultaneously re-evaluated by two oral mucosal specialists. The agreement between the general dentists and specialist diagnoses was low (54.8%) compared to a Swedish multicentre dental patient study, where 85% of the 803 cases were given the same diagnosis by the general practitioner and two oral mucosal specialists [2]. In our cohort, 20.9% of the mucosal changes documented were not given any primary diagnosis by the general dentists and were only classified by the specialists. This may be because the general dentist examiners were unfortunately not well trained in the diagnosis of oral mucosa but were better trained in classifying teeth and gingival diseases, which were examined during the same visit. The lack of biopsy may also hinder with our diagnoses, which is a point that should be taken into account in further investigations.

The prevalence of oral mucosal changes varies depending on the published study populations [1–6, 10, 17–28]. For example, in a recent evaluation of Slovenian citizens (2395 patients, aged 22–92 years) who attended a general dental practice, the prevalence of OMLs was 27% [3]. A similar result was found in an Italian study (4098 subjects, age range 19–96 years) where the prevalence of OMLs was 25.09% [25]. However, in a southern Indian study (1190 patients, age range 2–80 years) conducted in a specialist clinic, the prevalence of one or more OMLs was clearly higher, 41.2% [21]. In a large Chinese general population (n = 11,054) of 1- to 96-year-old inhabitants [1], the prevalence of OMLs (10.8%) was almost the same as in our cohort (10.5%), but less than in a Swedish study (6448 adults, mean age = 56.0), where OMLs were found in 14.7% of the participants [2]. The higher prevalence in the Slovenian, Italian, Indian and Swedish cohorts may also reflect the heavy use of tobacco in Slovenia and Italy, betel in India, and snuff in Sweden, which increase the incidence of OMLs.

Here, the ten most common mucosal findings were OLR, OLP, Fordyce granules, hyperkeratosis, fissured tongue, geographic tongue, fibroma, snuff-related lesions, amalgam pigmentation and median rhomboid glossitis. When comparing our results to other publications, similar lesions are often recorded, but their prevalence seems to vary depending on the study population [1–3]. Some studies have focused on analysing mucosal lesions that raise a risk of oral cancer, or are associated with risk habits, such as smoking or denture wearing [22, 23, 25, 27]. In our study, we did not compare the presence of OMLs to chronic irritation, like dentures, which would have been worth conducting. The population reports have often focused on the prevalence of OMLs in adults of all ages including sometimes children sometimes not [1, 3, 17, 19, 27], therefore, it is not possible to directly compare results of our cohort of 45–47-year-olds with those.

Of normal variations, we detected mostly Fordyce granules, in 1.2% of the participants, whereas in the Chinese all-aged general population, the prevalence was only 0.5% [1], and in the Swedish adult study Fordyce granules were not listed [2]. generally, the prevalence of Fordyce granules has been higher than in our cohort: in Slovenia 1.9% [3], in Turkey 2.8% [19] and in India 6.6% [21] of the study population.

The most common OML was a white lesion group (6.5%) including OLP, OLR, hyperkeratosis, snuff-related lesion, leukoplakia and candidiasis, found more often in males (8.1% vs. 5.1%). In a Turkish study [19], white lesions were found in only 2.2% of the 5000 consecutive 17–85-year-old patients, whereas ulcerated lesions were recorded most often (6.6%), which we recorded in only 0.2% of participants.

The prevalence of OLP was 1.5%, similar as in Italy (1.46%), but higher than in China (0.8%), Turkey (0.8%), India (1.3%), or a more recent study from Slovenia (1.1%) [1, 3, 19, 21, 25]. However, in an earlier Slovenian study, OLP was found twice as often as the more recent study, in 2.3% of the participants [5]. In Cambodia, 1.8% of 1319 individuals studied had OLP [27]. Overall, our result of the OLP prevalence seems to fit well within the various reports ranging from 0.8% to 2.3%. Of the OLP patients, 62% were females, and it affected 1.8% of all females and 1.2% of all males. In a Swedish study (of over 30-year-olds), OLP was also more frequent among females (2.2% vs. 1.6%) [26], and in Slovenia, OLP was up to twice as common in females (3% vs. 1.5%) [5]. Interestingly, in a Cambodian population, OLP was only detected in females [27], whereas there were no sex-related differences in the prevalence of OLP in a Turkish study [19]. These results indicate that OLP in most, but not all, populations seem to be more common among females.

When we compared OLP with OLR, the prevalence of OLD was 3.5%, which is higher than in the most recent Swedish study [2], as well as in the Italian study (2.4% and 1.75%, respectively). Our higher prevalence may also be due to some misdiagnoses based on clinical images only, without biopsies, which were taken in the Italian study to confirm the diagnoses [25].

We registered leukoplakia in 9 (0.5%) of the participants, of which 5 were current, 2 former and 2 non-smokers. The prevalence of leukoplakia in our study was in a similar range as in recent Swedish [2] and Slovenian [3] studies where leukoplakia was found in 0.4% and 0.5%, respectively. However, in the Swedish study from 30 years ago, the prevalence of leukoplakia was higher (3.6%), and most of them (2.9%) were associated with tobacco [17]. In a recent Indian study, leukoplakia was detected in 5.7% of the 300 participants [27]. There are two studies from Italy in which the prevalence of oral leukoplakia varies significantly; in the randomly selected male participants over 40 years of age (n = 118) from northern Italy [10], the prevalence of leukoplakia was 13.8%, whereas of the 4098 male and female participants in the Turin area, leukoplakia was registered in 1.15%. In both studies, leukoplakia was more common in current smokers than in never smokers [10, 28]. Since smoking is more popular in India and Italy than in Finland currently, that can explain the higher number of leukoplakia lesions in those populations.

Smoking in Finland has decreased during the last decades, and in 2018, of the 20–64-year-old population, 14% were daily smokers [29], but in this northern Finland cohort 18% were still active smokers. The three most common lesions in smokers were OLP, hyperkeratosis and OLR. Current smokers had also more OLP than former or non-smokers, and current female smokers had OLP more often than males (5.7% vs. 1.8%). In Slovenia, OLP was diagnosed in only 1.5% of smokers (n = 392) [3], whereas in our study OLP was more than twice as common in current smokers (3.8%). Interestingly, for some reason geographic tongue was significantly more common in non-smokers (1.5%) than in current smokers (0.6%), similar to that seen in a Swedish study where 6.8% of non-smokers and 1.7% of smokers had a geographic tongue [30].

The use of snuff is lower in Finland compared to Sweden, but it has recently increased, especially among the youth. Although snuff is not sold in Finland, Swedish snuff is brought to Finland from Sweden. Based on the Finnish National Institute for Health and Welfare statistics 2018, about 5% of Finnish 20–64-year-old males and 0–1% of females used snuff daily [29]. In our cohort, the number of snuff users was 3.7% (n = 70), 44% used it regularly, and 2 were females. The three most common lesions in snuff users were snuff-related local lesions (14.3%), Fordyce granules (5.7%) and OLR (2.9%). None of the snuff users had OLP or leukoplakia. In the most recent Swedish report, snuff dipper’s lesion was found in 4.8% of the study population, but in that study the snuff users other mucosal changes were not recorded [2].

## Conclusions

In conclusion, this study provides data about the prevalence of oral mucosal changes in a middle-aged northern Finland population cohort. Although it is difficult to compare prevalence rates from different studies, these results are mainly in line with data reported by previous studies. The fact that 4% of the OMLs found here can be grouped as a potentially malignant disorder emphasises the importance of routine inspections and follow-ups of OMLs. Moreover, the importance of risk factors (tobacco and snuff) in the pathogenesis of mucosal changes is evidenced.

## Data Availability

The data used here are not available in order to protect participants’ identity.

## Abbreviations

CI: Confidence interval
CTL: Connective tissue lesions
NFBC: Northern Finland Birth Cohort
OLD: Oral lichenoid diseases
OLP: Oral lichen planus
OLR: Oral lichenoid reactions
OR: Odds ratio
SRL: Snuff-related lesions
